# Comparative Study of the Productive Parameters of Two Breeds of the *Bombyx mori* Silkworm Fed *Rhodotorula glutinis* Yeast

**DOI:** 10.3390/insects16050482

**Published:** 2025-05-01

**Authors:** Mihaela Hăbeanu, Anca Gheorghe, Nicoleta Aurelia Lefter, Mihaela Dumitru, Smaranda Mariana Toma, Petru Alexandru Vlaicu, Teodor Mihalcea

**Affiliations:** 1 Research Station for Sericulture Baneasa, 013685 Bucharest, Romania; anca.gheorghe@scsbaneasa.ro (A.G.); nicoleta.lefter@scsbaneasa.ro (N.A.L.); teodor.mihalcea@scsbaneasa.ro (T.M.); 2 National Research Development Institute for Animal Biology and Nutrition, Calea Bucuresti 1, 077015 Balotesti, Romania; mihaela.dumitru@ibna.ro (M.D.); smaranda.pop@ibna.ro (S.M.T.); alexandru.vlaicu@outlook.com (P.A.V.)

**Keywords:** *R. glutinis*, mulberry leaves, silkworm, larvae, cocoons

## Abstract

In this study, a comparative approach was used with the aim of filling the knowledge gap regarding the productive and economic parameters of two native *B. mori* silkworm breeds (*Lines C* and *Z*) fed with different diets, namely mulberry leaves alone and mulberry leaves with *R. glutinis* yeast at two different concentrations (1 × 10^9^ and 1 × 10^7^ CFU/mL). The trifactorial experiment was carried out with 600 silkworms during the fifth instar, with 300 for each breed and 50 larvae per rearing tray. The larvae were randomly assigned into three feeding groups, with two replicates: in the control group (C), the larvae received ordinary mulberry leaves; in the first experimental group (RG-1), the larvae were given mulberry leaves supplemented with *R. glutinis* yeast at a 1 × 10^7^ concentration; and in the second experimental group (RG-2), the larvae received the same quantity of mulberry leaves as the C and RG-1 groups, but with *R. glutinis* yeast at a 1 × 10^9^ concentration. *Line C* larvae in the RG-1 group revealed higher values for most of the productive traits assessed. There was a positive and significant correlation between the weights and lengths of the larvae and the silk gland, the silk gland and the shell, the cocoon and the pupae and the shell, and the shell and the pupa size (*p* < 0.001). The steady decline in the parameters at a higher concentration suggests that *R. glutinis* positively influences a number of silkworm growth features at a recommended dosage of 1 × 10^7^; however, further research is required to more precisely identify the mechanisms involved.

## Simple Summary

As a promising approach to strengthening the health and performance of *Bombyx mori* silkworms, microorganisms have features that can enhance the nutritional value of conventionally used mulberry leaves by stimulating growth, productivity, and disease resistance. To this end, *Rodotorula glutinis* yeast has unquestionable advantages, such as the ability to generate a variety of industrially valuable metabolites, including proteins and lipids, while being important for health via fatty acids, carotenoids, and enzymes. Although studies on animals have established the effectiveness of these yeasts, research has also revealed that they can have negative effects on immunocompromised individuals, which has led to controversies regarding their use. Less known as a mode of action in insects, *R. glutinis* demonstrates the important ability to beneficially impact growth and economic performance. In this study, we show that adding *R. glutinis* yeast to the diet of silkworms strengthens the effect of mulberry leaves, which leads to superior results in the *B. mori* silkworm, particularly in *Line C*, although a decrease was observed after a certain point.

## 1. Introduction

Insects have a meaningful connection to our lives [1]. Lepidoptera, the largest group of plant-eating insects, encompass a diverse range of species with different dietary preferences. For thousands of years, the domesticated silkworm, *Bombyx mori*, has been invaluable in the production of silk, as well as in the food industry and medicine. This is due to the numerous ways the by-products generated throughout its metamorphosis, from egg to larva, pupa (chrysalis), and adult moth, can be utilized. The *B. mori* L. life cycle is regarded as one of the most advanced forms of metamorphosis.

In addition to the challenges posed by high-density rearing conditions, insects also face deficiencies in nutrients and increased susceptibility to infections due to oxygen deprivation and high temperatures [2,3,4]. The monophagous feeding preference of *B. mori* is one of its primary traits, and the feed source for silkworm larvae consists almost exclusively of fresh mulberry leaves (*Morus alba* L.) [5]. The nutritional content and functional qualities of mulberry leaves have been enhanced over time by using contemporary methods in order to increase silkworm cocoons’ economic worth.

Previous studies have mostly concentrated on characterizing insect-associated yeast populations that are pertinent to human activities (either for production or as pests). However, understanding the relationships between yeasts and other insects more broadly is a crucial first step in improving our comprehension of ecological and evolutionary interactions [1].

Numerous studies have demonstrated that giving silkworms microbial supplements can lead to notable results [6]. Esaivani [7] and Savio et al. [4] described the possibilities and benefits of mixing mulberry leaves with specific probiotics and offered a helpful examination of probiotics used for silkworms, with the aim of improving their performance and resistance to natural diseases.

According to Chen et al. [8], *Lactobacillus* is the most frequently investigated probiotic used for insect feed. Commercial *Bacillus* and *Pseudomonas* biocontrol agents have also been developed. In 2020, mulberry leaves (*Morus nigra*) were mixed with varying concentrations of soybeans and *Saccharomyces cerevisiae* yeast to enhance silkworm cocoon characteristics and to evaluate their impacts on fecundity and fertility. The probiotics *Bifidum* and *S. cerevisiae* both markedly enhanced silk filament and cocoon qualities, with *Bifidum* resulting in a greater impact [9].

*Rhodotorula glutinis* (*R. glutinis*) is a species which belongs to the Basidiomycota phylum Sporodial order of the Urediniomycetes class [10,11]. This yeast represents a significant group of oleaginous microorganisms that undoubtedly possess numerous benefits, including their capacity to produce a wide range of industrially valuable metabolites, such as enzymes, lipids (at a rate of approximately 70 *w*/*w* of its biomass) with specific compositions of fatty acids, proteins, and carotenoids. The yeast *R. glutinis* has the ability to grow on natural, low-cost substrates, such as diverse raw materials and industrial waste (e.g., molasses and cellulose hydrolysates etc.), which is clearly beneficial for the production of metabolites [11,12]. Numerous investigations into the technological applications of *Rhodotorula spp.* yeast have been published in recent years [10,13,14]. This microorganism is thus considered a promising option for the microbial production of polyunsaturated fatty acids and as a high-enthalpy nutrient for aquaculture feed [15]. Furthermore, *R. glutinis* is capable of synthesizing numerous minerals in various quantities [16].

Although *R. glutinis* was once considered to be non-pathogenic, recent studies have shown that it is an opportunistic parasitic pathogen that can colonize and infect human patients with compromised immune systems [17,18]. The pathogenicity of *Rhodotorula* spp. in animals has also been reported in a few outlying cases. Most human *Rhodotorula* infection cases were caused by fungemia associated with central venous catheter (CVC) use.

Although *R. glutinis* has been classified as a pathobiont for specific species under particular circumstances, its pathogenicity to insects has not been proven. As far as we are aware, there is little information on the potential of *R. glutinis* to harm insects or on its use on insects. A possible explanation for the less relevant pathogenicity potential in insects would be that insects differ greatly from other species in their physiology and structure, and in their quick moulting processes and metamorphoses. As far as we know, no prior research has demonstrated that the *R. glutinis* was used for *B. mori* silkworms or other insects.

It is unclear whether the gut is the primary site of all opportunistic infections, as Hof [19] discussed. The significant presence of these yeasts in the gut suggests that the equilibrium of the microbial flora changed due to a variety of reasons. However, when these *Rhodotorula* spp. yeasts colonize the host organism, they can have beneficial effects on health, as they produce a significant number of useful nutrients. In addition, probiotic effects can be identified as a result of the regulation of pathogenic bacteria multiplication and through the neutralization or destruction of their toxins [19].

The hypothesis of the present study posited that *R. glutinis* yeast would enable silkworms to achieve their productive potential without compromising their health. In accordance with current research practices, this study was carried out using a comparative approach to examine the productive parameters of two native silkworm breeds (*Lines C* and *Z*) that have been linked to controlled feeding (mulberry leaves with or without *R. glutinis* yeast at two different concentrations (1 × 10^9^ and 1 × 10^7^ CFU/mL).

## 2. Materials and Methods

### 2.1. Rearing Conditions and Biological Material

A trifactorial experiment (2 × 3 × 3; lines × diets × time) was carried out in the period of May–June 2024, at Baneasa Station (44°29′33″ N 26°04′45″ E), situated in the north of Bucharest, Romania.

In order to assess the productive parameters of the larvae and cocoons, two indigenous monovoltine *B. mori* silkworm breeds were chosen (*Line Z* and *C*). The biological material was obtained from the Romanian breeding stock center, SCS Baneasa, Bucharest, which has provided “Romanian” breeds for more than 10 years. The breed *Line Z* was created through infusion crossing of the White Chinese 29 breed (originating in China) and the Tokay breed (originated in Japan), and the breed *Line C* was obtained by crossing White Cislau (originating in Romania) and Tokay. These breeds were crossed by infusion for several generations in order to obtain useful traits. *Line Z* breed has gray-green eggs and yellow chorions, larvae have larval signs, and cocoons are belted and oval. The *Line C* breed is characterized by gray-green eggs and white chorions, larvae predominantly have an integument which lacks larval signs, and cocoons are unbelted and oval.

These breeds can be raised in Romanian environmental conditions during appropriate seasons (May–June).

Microscopic analysis revealed that the eggs were disease-free, and the larvae were subsequently observed throughout their phases of metamorphosis.

### 2.2. Experimental Design

**Figure 1:**
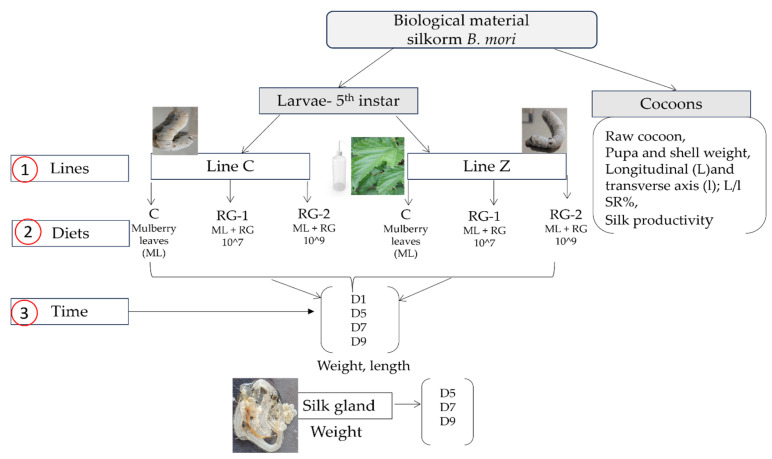
Experimental design. Legend: *B. mori*—*Bombyx mori*; biological material—breeds *Line C* and *Line Z*; diets—C (control group), RG-1 (experiment group 1, fed with mulberry leaves (ML) + *Rhodotorula glutinis (R. glutinis)* at a 1 × 10^7^ concentration), and RG-2 (experiment group 2, fed with mulberry leaves + *Rhodotorula glutinis (R. glutinis)* at a 1 × 10^9^ concentration); D—day.

During the 5th instar, 600 disease-free *B. mori* silkworms (300 each breed, with 50 larvae per rearing tray) were raised in hygienic standard conditions (24–26 °C and 70–80% relative humidity) and were randomly placed into three feeding groups, with two replicates each. In the control group (C), larvae received only standard mulberry leaves; in the first experimental group (RG-1), larvae were given mulberry leaves supplemented with *R. glutinis* yeast at a 1 × 10^7^ concentration; in the second experimental group (RG-2), larvae received the same quantity of mulberry leaves as the C and RG-1 groups, combined with *R. glutinis* yeast at a 1 × 10^9^ concentration (Figure 1). Previous studies that aimed to test different concentrations of probiotics were the starting point in establishing the yeast concentration for this study [6,7,8,9].

For each replicate, we used carton trays (30 × 15 × 5 cm) that were positioned next to one another, although still sufficiently separated, in order to ensure similar environmental conditions while preventing the mixing of larvae from various trays.

Every day, the litter was collected from the larvae’s rearing bed.

The following parameters were evaluated: the weight and length of the larvae, the weight of the silk glands, the weight of the cocoon, the weight of the pupa and shell, the longitudinal (L) and transverse (l) axes and their ratios, the proportion of the shell, and the productivity of the larvae.

### 2.3. Feeding

Every morning, between 8:00 and 9:30 a.m, the larvae belonging to C group were fed with 100 g of mulberry leaves (50 g per replicate).

In the first nine days, for the larva feed, 20 mL of a solution consisting of distilled water and yeast (at a concentration of 1 × 10^7^ for the RG-1 group or 1 × 10^9^ for the RG-2 group) was sprayed onto 100 g of mulberry leaves (50 g per replicate) at room temperature every morning between 8:00 and 9:30 a.m. The sprayed mulberry leaves were left for 20 min before being dried. The larvae in the control group received only mulberry leaves.

### 2.4. In Vitro Testing of R. glutinis

*R. glutinis* yeast was obtained from the Culture Collection of Yeast (020-002-033 CCY, Bratislava) and was kept in the Laboratory of Animal Nutrition and Biotechnology’s internal collection, Romania. Here, the in vitro testing process is described briefly: the yeast was activated for 24–48 h at 28 °C and 150 rpm under aerobic conditions and were sub-cultured at least three times in yeast–peptone–dextrose (YPD) broth medium (Himedia M1365). The YPD broth, at a 1:10 ratio (*wt*/*v*), comprised 2% peptic digest of animal tissue, 1% yeast extract, and 2% dextrose dissolved in distilled water. The pH was adjusted to 6.5 ± 0.2, followed by sterilization at 121 °C for 15 min. After incubation, the purity of the yeast culture was verified using YPD agar plates. The physiological characteristics of *R. glutinis* were evaluated and the morphology of the colony was taken into consideration. The optical density (OD) at 600 nm colony-forming units (CFU) per mL was determined in triplicate. The CFU/mL was 2.8 × 10^11^ and the OD600 nm was 2.095.

Macroscopically, the colonies of *R. glutinis* grown on solid culture medium were soft in consistency, with regular borders and a mucilaginous appearance. A distinctive feature of *R. glutinis* is its red/pink color, occurring due to the production of carotenoids. As for the size of the colonies, they can vary in size, forming visible colonies within a period of 24–48 h at 28 °C, which are oval or round in shape and are approximately 0.5–1.3 mm in diameter.

Microscopically, the cells of *R. glutinis* are Gram-positive. They were colored purple as a result of Gram staining and were small and oval in size, with most of them being in different stages of budding.

For the following tests, the initial viable cell counts of 11.45 Log10 were adjusted to about 1 × 10^9^ CFU/mL and 1 × 10^7^ CFU/mL. The percentage of protein was 36.95%.

### 2.5. Chemical Analyses

The feed chemical composition was determined in duplicate at IBNA Balotesti’s Chemistry Laboratory (Table 1). The gross chemical composition was ascertained using standardized procedures in accordance with European Commission (EC) Regulation 152/2009. In short, the semiautomatic traditional Kjeldahl method (Auto 1030 Analyzer, Tecator Kjeltec, SR EN ISO 5983-2, 2009) was applied to assess crude protein. Following standard SR ISO 6492 (2001), the above-mentioned Regulation (EC) no. 152/2009 (sampling and analytical methods for the official inspection of feeds), and ISO standards, the fats (ether extractives) were determined via extraction in organic solvents. The cellulose was also assessed using an intermediate filtering process under the previously mentioned European Commission (EC) Regulation no. 152 (2009) and the standard SR EN ISO 6865/2002, and ash (ISO 2171/2010) and dry matter (ISO 6496/2001) were determined using the gravimetric method.

### 2.6. Measurements of Silkworm Larvae and Cocoons

The production parameters for each individual larva were established according to breed and feeding group.

Twenty larvae per group (60 per breed and 10 per replication) were chosen at random during the 5th instar to determine larva characteristics (weight, length, average weight gain) and they were measured on the first day and ninth day.

The growth index was calculated using Rahmathulla et al.’s equation [20]:

Growth index = final weight of the larvae (g) − initial weight of the larvae (g)/initial weight of the larvae (g).

In order to determine the dynamic changes in the dependent variables, evaluations were carried out on the first, fifth, seventh, and ninth days.

To test whether the addition of *R. glutinis* yeast altered the weight of the silk glands, on the fifth, seventh, and ninth day, before spinning, four larvae per group (twelve per breed and two of each replicate) were dissected for silk gland extraction.

After spinning, twenty cocoons (ten per duplicate) were chosen at random, collected individually, and had their floss removed. The weights of the raw cocoon, the pupa, and shell, as well as the longitudinal (L) and transverse axes (l), were determined after the cocoons were carefully opened with a cutter to extract the pupae. Measurements were carried out using a digital caliper and an electronic scale. To obtain the shell ratio, the formula of Sekar et al. [21] was used as follows: S.R.% = weight of cocoon shell/weight of cocoon × 100. The formula of Saranya et al. [22] was utilized to obtain silk productivity values (cg/day): shell weight/duration of 5th instar (9D).

### 2.7. Statistical Analyses

The data are presented as means and standard errors of the means (SEMs). The software IBM SPSS, version 20.0 (SPSS Inc., Chicago, IL, USA), and the general linear model (GLM) multivariate test were used to statistically analyze the collected data. The Shapiro–Wilk test was used to assess the data distribution. The differences between the means were evaluated using the least significant difference (LSD) test. The specific effects and significances of the individual breed and diets, as well as interaction between diet × breeds, were determined. Silkworm breeds and applied treatments were regarded as the fixed influencing factors. Each larva and each cocoon were considered experimental units. Means were considered highly and significantly different if *p* values were less or equal to 0.01, 0.001, or 0.0001; differences were considered significant if *p* ≤ 0.05, and the probability that the treatment or breed affected the results was considered to be 0.05 < *p* > 0.05.

The repeated measurements test, GLM, was used to statistically test the variations in the parameters at different time points. The association between different variables was evaluated using the Pearson correlation, and the Akoglu [23] guide was used to interpret the correlation coefficients.

Regression analysis was utilized to evaluate the degree of association with the predictor.

The charts were generated using the statistical software SPSS.

## 3. Results

### 3.1. Larva Productivity Parameters

The feed component intakes are given in Table 2.

As expected, the *Line C* breed exhibited a higher feed intake. For *Line C* breed, the intake was superior in RG-2, but for *Line Z*, RG-1 had the higher intake (>25% higher total daily DM intake in *Line C* than *Line Z*).

Using a combination of fixed effects, Table 3 displays the descriptive statistics of the assessed characteristics (weight, length, and silk gland weight) for each breed with respect to the various feeding groups, as well as for diet × breed interactions.

Regardless of the parameters, the *Line C* breed exhibited superior values at day nine (*p* < 0.0001) compared to *Line Z* breed: the weight was 25.54% higher, the WG was 23.63% greater, the length was 8.55% greater, and the silk gland weight was 34% higher.

Overall, the larva weights were 4.07 g for *Line C* and 3.37 g for *Line Z*. The data showed that the larval weight varied between 0.63 and 6.56 g throughout the whole period, with RG-1 and RG-2 treatments demonstrating a greater effect. However, as the data in Table 4 show, the growth index was greater in *Line Z* breed (overall higher at 7.3%), with the RG-2 group having the highest value. The minimum index growth value was observed in the control group and in *Line C*. This indicates a higher initial larva weight in *Line C* breed.

As indicated by the data, the silkworm larva lengths ranged from 33.48 to 80.95 mm, with groups additionally fed *R. glutinis* yeast exhibiting the greatest impact.

The larvae in the RG-1-fed group were more significantly affected in terms of weight (>1.05 times that of the control group), whereas the larvae in the RG-2 fed group were more significantly impacted in terms of length (>1.01 times that of the control group).

The effect of the RG-1 treatment on the silk gland was more noticeable (>14.56% in the RG-1 group compared to the control group).

Breed and diet did not appear to have a compounding effect, and these two influencing factors showed no significant interaction (*p* > 0.05).

No replication influence was observed, regardless of the factors evaluated (*p* > 0.05).

**Figure 2:**
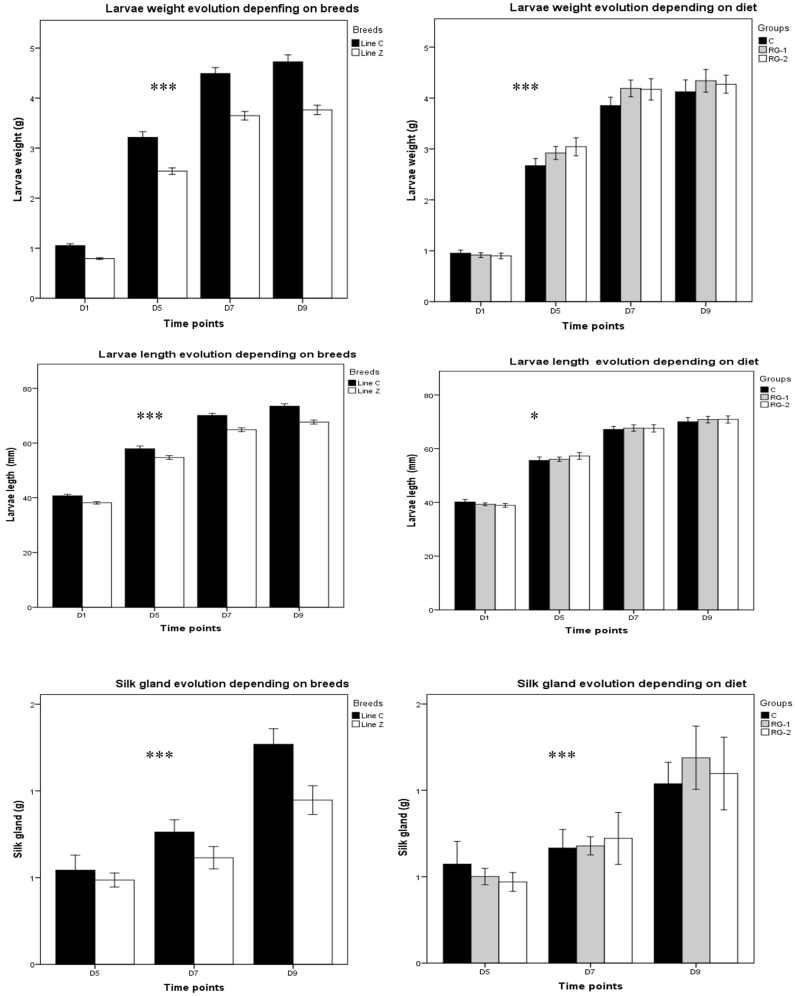
Larva and silk gland characteristics at different time points. Biological material—breeds *Line C* and *Line Z*; diets—C (control group), RG-1 (experiment group 1, fed with mulberry leaves + *Rhodotorula glutinis (R. glutinis)* at a 1 × 10^7^ concentration), and RG-2 (experiment group 2, fed with mulberry leaves + *Rhodotorula glutinis (R. glutinis)* at a 1 × 10^9^ concentration); D—day. *** The mean difference was highly significant at the *p* < 0.0001. * The mean difference was significant at the *p* < 0.05 level.

Larva and silk gland growth evolution for the 5th instars were significantly boosted at various time points (Figure 2). On day nine, the weight of *Line C* larvae was 4.49 times higher than on day one, 1.47 times higher than on day five, and 1.05 times higher than on day seven. For *Line Z*, weight was 4.75 times higher on day nine than on day one, 1.06 times higher on day seven, and 1.48 times higher than on day five. Overall, *Line C* weight was 1.25-fold higher compared to *Line Z*.

The trend for the length parameter was similar to the weight parameter: for *Line C*, on day nine, the larvae were 1.80 times longer than on day one, 1.27 times longer than on day five, and 1.04 times longer than on day seven; for *Line Z*, on day nine, larvae were 1.77 times longer compared to day one, 1.24 times longer than on day five, and 1.04 times longer than on day seven. Overall, the length parameter saw a 1.07-times increase in *Line C* vs. *Line Z.*

The repeated measurement test in GLM revealed higher significant variations (*p* < 0.0001) of the larvae and silk gland weight at various time points and significant differences in the larvae length as diet effects at different time points (*p* < 0.02). The time effect was particularly noticeable in breed *Line C*. Specifically, the silk gland on day nine was 1.66 times larger than one day seven and 2.35 times larger than on day five. For *Line Z*, on silk gland was 1.44 times larger compared to day seven and two times larger than on day five.

Overall, compared to *Line Z*, the breed *Line C* had 1.35 times higher silk gland weight on day nine.

By applying the multiple linear regression model, our results show that the length of the larvae can be considered a potential predictor for the size of the silk gland (β coefficient = +0.76, R^2^ = 0.59, *p* < 0.0001). The linear model is y = ax + c, where y is the silk gland size (dependent variable), a is the slope, x is the larva length (independent variable), and c is the intercept.

Silk size = (64.53 × 0.037) − 1.63.

### 3.2. Cocoon Economic Characteristics

Table 5 summarizes the average value of cocoon traits depending on the breed and diet. The interactions between these factors were also considered.

Overall, the weights of the cocoons varied from 1.59 to 2.66 g, the shell weights ranged from 0.31 to 0.55 g, the pupa weights varied from 1.22 to 2.11 g, and the silk productivity ranged from 3.44 to 6.11 cg × d^−1^. However, regardless of the cocoon traits, the *Line C* values were superior. This can be seen as follows: in *Line C*, the pupae weighed 8.55% more, the cocoons weighed 11.98% more, and the shells weighed 26.5% more. Consequently, the SP increased by 22.7% and the SR increased by 12.91%.

The majority of the variables assessed were positively affected by the RG-1 treatment. Accordingly, the maximum mean values were observed in the *Line C* breed RG-1 group (cocoon weight, 2.337 g; shell weight, 0.487; and pupa weight, 1.837 g), yielding a higher SR% value, while the minimum values (20.941%) were observed in the *Line Z* breed RG-2 group (cocoon weight, 1.847 g; shell weight, 0.332 g; and pupa weight, 1.505 g), which led to a decrease in the SR% to 18.136. No interaction between genetics × diet was observed (*p* > 0.05).

### 3.3. Interrelation Between Parameters

The Pearson correlation was utilized to verify the correlation between larva and cocoon parameters (Table 6). A strong, positive, and highly significant correlation was observed between the weights and lengths of larvae (R = 0.82) and cocoon and pupa weights (R = 0.981), and a moderate, highly significant correlation was observed between larva and silk gland weights (R = 0.601), silk gland and shell weights (R = 0.622), cocoon and shell weights (R = 0.694), and shell and pupa weights (R = 0.552).

## 4. Discussion

In recent years, microbial technology has attracted considerable attention from scientists due to its applicability in many different industries, most notably in the improvement of productivity and health or as a component in new dietary additions. According to Suraporn et al. [24], the microorganisms *Bifidobacterium*, *Lactobacilus* spp., *Burkholderia cepacian*, and *Bacillus* spp. were utilized to provide benefits against diseases, such as improved rates of development and resistance. *Saccharomyces cerevisiae* yeast has been shown, in numerous studies, to have positive effects on silkworms [9,25].

Similar to other species, the growth performance and development of *B. mori* L. silkworms are influenced by both nutritional efficiency and genetic potential.

This study was motivated by the need to fill the information gap surrounding the production performance of certain silkworm breeds that are currently being bred by sericulturists in Romania. Therefore, the present investigation evaluates how fortifying mulberry leaves with two distinct *R. glutinis* yeast concentrations affects the productivity characteristics of *Line C* and *Line Z* breeds of *B. mori* silkworms.

To our knowledge, there was no previous evidence that this yeast has specific applications for *B. mori* silkworms or any other insects. Our research demonstrates that while the bioactive substances in yeast may have a positive effect on specific productivity characteristics, higher concentrations do not always have a more significant effect than lesser concentrations or can even result in a decrease.

Since *R. glutinis* has been shown to have both beneficial and harmful effects on humans, it is regarded as a controversial microorganism. *R. glutinis* yeast has the capacity to produce a variety of beneficial compounds, including microbial lipids, polyunsaturated fatty acids, proteins, pigments, and enzymes. Although *R. glutinis* yeast has been shown to colonize and infect immunocompromised individuals, transforming them into opportunistic pathogens, there are, as far as we are aware, no data in the literature concerning its possible adverse effects on animals or insects.

Since 1999, when Arras et al. [26] demonstrated that *R. glutinis* is not toxic, a number of studies have been conducted to assess its effects on poultry [27,28] and pigs [29] or its potential as a source of industrial pigments and industrial bioactive compounds [10,13]. According to Sun et al. [30], hens’ laying performance and egg quality were enhanced by the dietary addition of *R. glutinis* yeast. Hu et al. [29] found that the growth performance of piglets increased as a result of the oral administration of *R. glutinis*, which also maintained the intestinal microbial balance of the animals and boosted their antioxidant and gastrointestinal digestion capacities. Xu et al. [27] highlighted that *R. glutinis* had the ability to metabolize a variety of materials as a source of carbon and nitrogen. In addition, they demonstrated that, in the production of carotenoids and other nutrients, *R. glutinis* improved tofu whey wastewater utilization, increased the wastewater’s utilization value, and diminished resource waste and adverse environmental impacts [27]. Concerning insects, yeasts are an important source of feed. As outlined by Stefanini [1], our understanding of how insects and yeasts interact is still developing. Today, it is known that these relationships are important for the behavior and health of the host organism as well as for proliferation of yeast in the surrounding environment. However, we still do not fully understand the mechanisms that control these interactions or how they impact the lives of microbes and animals.

Multiple variables, including feed formula, environment, and silkworm breed, can affect yeast performance. In this study, 5^th^-instar silkworms were fed mulberry leaves. The adaptation between breeds may have been a result of varying diets. The reasons behind the specific differences in digestion rates of the three groups studied in this work need further investigation. Research conducted by Samami et al. [31] demonstrated that the digestive efficiency of silkworms is controlled by genetics. According to Hemmatabadi et al. [32], the genetic potential of each species, its environment, and the interactions between these two factors define a silkworm’s production capability. In present paper, we observed that the addition of *R. glutinis* to the diets of silkworm larvae from two distinct breeds (*Lines C* and *Z*) had a more noticeable impact on the productive indicators Line C larvae (weigh, length, and WG). Genetic factors were mostly responsible for the larvae’s steady development during the fifth instar.

Other characteristics include the capacity to consume feed from a variety of sources and a heightened resilience to disease. In our case, the *Line C* RG-2 larvae demonstrated a higher dry feed intake, while *Line Z* larvae had a dry intake closer to that reported by Tanjung et al. [33] in standard environmental conditions. We found that adding yeast, at any concentration, improved the nutritional value of mulberry leaves, especially in RG-2 diets, which had a favorable impact on certain parameters. However, this was not always reflected in the results. One explanation of this could be potential toxicity above a certain threshold, even if this had no impact on day-to-day health, most likely because exogenous yeast interacts associatively with the host microbiota. It seems reasonable to assume that the dietary addition of *R. glutinis* yeast to the diets of silkworm larvae might stop an infectious cycle rather than increase the risk of disease. Furthermore, due to successive moulting, certain tissues are removed during silkworm growth; this boosts the immune system and significantly affects yeast populations. On the other hand, although higher concentrations of yeast increase dietary protein and fat contents, it is possible that they can lead to an excess of these nutrients, resulting in a decrease in performance when compared to a diet with a lower content of yeast. However, in the Line Z RG-1 group, the protein intake was higher than in the RG-2 group. Regardless, the complex nature of the interactions between the intestinal microbial communities of silkworms has not been documented previously.

Given these factors and the comparatively brief dosing period, it is possible that *R. glutinis* generally has a beneficial rather than detrimental impact on the characteristics of silkworm larvae; however, an appropriate concentration is crucial.

It is well known that the nutritional value of mulberry leaves and the growth of larvae are key elements in silk production [34]. The growth, development, and survival of the examined larvae were impacted by the nutritional value of the leaves, which is in line with the findings of Babirye et al. [35]. Larvae have differing responses to various treatments, which can include modifications to their feed; the time point at which they are fed; levels of consumption, metabolism, and enzyme synthesis; and changes to nutritional and physiological processes.

Numerous studies on the weight and length of the larvae, growth rate, cocoon weight, pupa weight, and cocoon shell weight have demonstrated the significance of the correlations between larva traits in silkworm breeding [32].

We demonstrated that the silkworm larval growth in the experimental and control groups increased substantially from the first day to the ninth day. Between days 7 and 9, the growth rate slowed as the larvae began to prepare for the spinning phase of their metamorphosis.

Our findings were comparable to those reported by Muzamil et al. [36]. In their study, 5^th^-instar silkworms were categorized into a control group that was fed fresh mulberry leaves and groups 2–15, which were regarded as experimental groups and fed mulberry leaves coated, with amino acids as their initial meal. Alipanah et al. [37] assessed and compared the performances of the silkworms fed with three different types of mulberry leaves. The authors of this article demonstrated the significance of mulberry leaves’ nutritional value, which is dependent on their variety.

Silk filament length and cocoon quality are influenced by the weight of the silk gland. In the current experiment, the weight of the silk gland was significantly influenced by genetics (*Line C* exhibited higher mean values) and diet (the RG-1 group had a higher weight compared to the control group). The Pearson correlation demonstrated that the silk gland is positive and highly significantly correlated with larva growth rate and shell weight. We established a model that demonstrated that the size of the silk gland can be accurately predicted by the length of the larvae.

Cocoon and shell weight are the two most important parameters to be taken into account when evaluating productivity. Similarly to Mirhosseini et al. (2010), who were cited by Hemmatabadi et al. [32], in our research, we found a significantly positive Pearson correlation between cocoon weight and cocoon shell weight.

Regardless of the breeds or diet, the cocoon properties (cocoon, shell, and pupa weight) obtained in our trial were superior to those reported by Tanjung et al. [33] in normal environmental conditions and by Ziaeddin et al. [38], who examined five native Iranian and two commercial silkworm lines.

According to Rahmathulla et al. [20], fortifying mulberry leaves with additional nutrients is a viable modern technique to increase a cocoon’s economic value. Our research, through certain indicators, revealed that a smaller yeast concentration could yield better results. At higher concentrations, it is possible that the balance of the microbial flora in a silkworm’s gut is disturbed.

Based on our findings, we can speculate that *R. glutinis* treatment has an important impact. Therefore, on the one hand, these yeasts produce a lot of beneficial nutrients, including proteins, lipids, and carotenoids, etc. On the other hand, *R. glutinis* may have a probiotic effect on the larvae’s digestive tract by controlling the proliferation of pathogenic microbes and neutralizing or destroying toxins.

In the standard environmental condition, we can assume that yeast can act by manipulating the gut microbiota profile, improving the physiological activities of the host and capacity to combat disease, which leads to a better utilization of the nutrients and energy.

## 5. Conclusions

In this study, for the first time, we outlined the comparative data of the productive parameters between two breeds of the *B. mori* silkworm, which demonstrates the effects of *R. glutinis* as a supplement for mulberry leaves.

The results of the present investigation showed that *R. glutinis* yeast, unknown as a supplement for silkworm feed, has a positive impact on growth and economic parameters. Rich in bioactive compounds, *R. glutinis* added value to mulberry leaves.

In our study, genetics was the most critical factor that affected silkworm characteristics. The effects were more pronounced at a 1 × 10^7^ concentration, while above this threshold, the impact on certain parameters was less evident; nevertheless, further research is required to more precisely define the dosage amount and the mechanisms involved.

## Figures and Tables

**Table 1:** Chemical composition of diets (g × DM^−1^).

Items	Groups	DM g/100 g Fresh	OM	CP	EE	CEL	Ash
Feed	C	25.44	22.56	6.25	0.46	3.73	2.88
	RG-1	25.29	22.19	6.35	0.63	3.85	3.10
	RG-2	28.33	24.47	7.31	0.71	4.37	3.86

Abbreviations: DM, dry matter; OM, organic matter (DM-Ash); CP, crude protein; EE, ether extract; CEL, cellulose.

**Table 2:** Average daily feed intakes (g) of different silkworm groups.

Items	Line C			Line Z		
	C	RG-1	RG-2	C	RG-1	RG-2
DM	0.726	0.756	0.775	0.567	0.624	0.608
Protein	0.185	0.192	0.197	0.144	0.159	0.155
Lipids	0.066	0.068	0.070	0.051	0.057	0.055

Abbreviations: DM, dry matter. Legend: biological materials—breeds Line C and Line Z; diets—C (control group), RG-1 (experimental group 1, fed with mulberry leaves + *Rhodotorula glutinis (R. glutinis)* at a 1 × 10^7^ concentration), and RG-2 (experimental group 2, fed with mulberry leaves + *Rhodotorula glutinis (R. glutinis)* at a 1 × 10^9^ concentration).

**Table 3:** Effects of breed and diet on silkworm growth performance in the 5th instar.

Items		Wt—D1 (g)	Wt—D9 (g)	WG (g)	L—D1 (mm)	L—D9 (mm)	Silk gl. —D5 (g)	Silk gl. —D9 (g)
***Breed effect***								
Line C		1.051	4.724	0.408	40.717	73.457	0.543	1.269
Line Z		0.792	3.763	0.330	38.132	67.671	0.486	0.947
***Diet effect***								
C		0.951	4.122	0.352	40.127	70.010	0.573	1.037 ^a^
RG-1		0.914	4.339	0.381	39.268	70.795	0.501	1.188 ^b^
RG-2		0.898	4.270	0.374	38.877	70.887	0.470	1.098
***Breed*** × ***diet***								
Line C	C	1.103	4.696	0.399	42.045	73.597	0.663	1.155
	RG-1	1.017	4.821	0.424	39.948	73.465	0.500	1.357
	RG-2	1.032	4.655	0.403	40.158	73.309	0.467	1.295
Line Z	C	0.799	3.547	0.423	38.211	66.424	0.485	0.920
	RG-1	0.811	3.855	0.338	38.589	68.125	0.502	1.020
	RG-2	0.764	3.886	0.347	37.595	68.464	0.470	1.097
SEM		0.015	0.060	0.006	0.217	0.387	0.022	0.043
***p values***								
Breeds		<0.0001	<0.0001	<0.0001	<0.0001	<0.0001	<0.0001	<0.0001
Diet		0.383	0.330	0.137	0.054	0.601	0.107	0.050
Breed × diet		0.150	0.173	0.251	0.016	0.211	0.115	0.454

Means of 20 larvae per group and 10 larvae per replicate. Data were analyzed as 2 × 3 factorial designs. Means with different superscripts within a column differ (*p* < 0.05). Abbreviations: Wt—weight; L—length; WG—average weight gain; Silk gl.—silk gland; SEM—standard error of the mean. Legend: biological material—breeds *Line C* and *Line Z*; diets—C (control group), RG-1 (experiment group 1, fed with mulberry leaves + *Rhodotorula glutinis (R. glutinis)* at a 1 × 10^7^ concentration), and RG-2 (experiment group 2 fed with mulberry leaves + *Rhodotorula glutinis (R. glutinis*) at a 1 × 10^9^ concentration); D—day.

**Table 4:** Effect of breed and diet on index growth (g).

Breeds	Group	Index Growth
Line C	C	3.257
	RG-1	3.740
	RG-2	3.511
Overall		3.503
Line Z	C	3.439
	RG-1	3.754
	RG-2	4.086
Overall		3.760

Legend: biological material—breeds *Line C* and *Line Z;* diets—C (control group), RG-1 (experiment group 1, fed with mulberry leaves + *Rhodotorula glutinis (R. glutinis)* at a 1 × 10^7^ concentration), and RG-2 (experiment group 2, fed with mulberry leaves + *Rhodotorula glutinis (R. glutinis)* at a 1 × 10^9^ concentration).

**Table 5:** Effects of the breed and diet on cocoon characteristics for silkworms in the 5th instar.

Items		Cocoon Weight (g)	Shell Weight (g)	Pupa Weight (g)	Longitudinal Axes (L, mm)	Transversal Axes (l, mm)	L/l	SR%	SP (cg/day)
***Breeds effect***									
Line C		2.225	0.463	1.752	34.165	18.942	1.805	20.988	5.148
Line Z		1.987	0.366	1.614	33.998	18.312	1.859	18.588	4.074
***Diet effect***									
C		2.038	0.418 ^T^	1.617	34.086	18.555	1.837	20.709	4.652 ^T^
RG-1		2.243 ^T^	0.438 ^a^	1.797	34.490	18.865	1.833	19.546	4.875 ^a^
RG-2		2.035 ^T^	0.387 ^bT^	1.635	33.670	18.460	1.827	19.109	4.305 ^bT^
***Breed*** × ***diet***									
Line C	C	2.115	0.460	1.655	34.557	18.770	1.841	21.941	5.111
	RG-1	2.337	0.487	1.837	34.632	19.232	1.804	20.941	5.416
	RG-2	2.223	0.442	1.765	33.307	18.822	1.770	20.082	4.916
Line Z	C	1.963	0.377	1.580	33.615	18.340	1.834	19.477	4.194
	RG-1	2.150	0.390	1.757	34.347	18.497	1.861	18.152	4.333
	RG-2	1.847	0.332	1.505	34.032	19.097	1.884	18.136	3.694
SEM		0.054	0.012	0.047	0.242	0.155	0.020	0.473	0.137
***p values***									
Breed		0.020	<0.0001	0.151	0.739	0.040	0.188	0.008	<0.0001
Diet		0.211	0.014	0.244	0.405	0.560	0.979	0.378	0.014
Breed × diet		0.615	0.683	0.659	0.408	0.899	0.521	0.920	0.683

Means of twelve cocoons per hybrid, eight cocoons per group, and four cocoons per replicate. Data were analyzed as 2 × 3 factorial designs. Means with different superscripts within a column differ (*p* < 0.05). Abbreviations: SR—shell ratio; SP—silk productivity; SEM—standard error of the mean. Legend: biological material—breeds *Line C* and *Line Z*; diets—C (control group), RG-1 (experiment group 1, fed with mulberry leaves + *Rhodotorula glutinis* (*R. glutinis*) at a 1 × 10^7^ concentration), and RG-2 (experiment group 2, fed with mulberry leaves + *Rhodotorula glutinis* (*R. glutinis*) at a 1 × 10^9^ concentration); D—day.

**Table 6:** Pearson correlation for interactions between parameters.

Items	Pearson Correlation/ *p* Value	Larva Weight	Larva Length	Silk Gland Weight	Cocoon Weight	Shell Weight	Pupa Weight
Larva weight	R	1.00	0.821 **	0.601 **	0.12	0.418 *	0.01
	*p* value		<0.001	<0.001	0.59	0.04	0.98
Larva length	R		1.00	0.590 **	−0.04	0.37	−0.15
	*p* value			<0.001	0.85	0.08	0.47
Silk gland weight	R			1.00	0.34	0.622 **	0.23
	*p* value				0.10	<0.001	0.28
Cocoon weight	R				1.00	0.694 **	0.981 **
	*p* value					<0.001	<0.001
Shell weight	R					1.00	0.552 **
	*p* value						0.01

R = Pearson correlation. Means with different superscripts within a column differ. ** *p* < 0.001 and *p* = 0.01, correlation is highly significant; * *p* < 0.05, correlation is highly significant.

## Data Availability

The data presented in this study are available upon request from the corresponding author.
